# Diagnostic accuracy of the BioFire® FilmArray® pneumonia panel in COVID-19 patients with ventilator-associated pneumonia

**DOI:** 10.1186/s12879-023-08486-4

**Published:** 2023-08-09

**Authors:** Gabriel Cojuc-Konigsberg, Alberto Moscona-Nissan, Alberto Guijosa, Christian D. Mireles Dávalos, María E. Jiménez Martínez, Mario A. Mújica Sánchez, Víctor F. Hernández Huizar, Martha A. Durán Barrón, Karen Villarreal Gómez, Regina Andrade-Galindo, Montserrat Ordóñez-Oviedo, Grecia Deloya Brito, Eduardo Becerril Vargas

**Affiliations:** 1 Clinical Microbiology Laboratory, National Institute of Respiratory Diseases, Mexico City, Mexico; 2 Health Sciences Faculty, Universidad Anahuac Mexico, Mexico City, Mexico; 3 School of Medicine, Universidad Panamericana, Insurgentes Mixcoac, Donatello 59, 03920 Mexico City, Mexico

**Keywords:** BioFire® FilmArray® Pneumonia Panel, Ventilator-Associated Pneumonia, COVID-19, Syndromic panels, Antimicrobial Resistance

## Abstract

**Background:**

Ventilator-Associated pneumonia (VAP) is one of the leading causes of morbidity and mortality in critically ill COVID-19 patients in lower-and-middle-income settings, where timely access to emergency care and accurate diagnostic testing is not widely available. Therefore, rapid microbiological diagnosis is essential to improve effective therapy delivery to affected individuals, preventing adverse outcomes and reducing antimicrobial resistance.

**Methods:**

We conducted a cross-sectional study of patients with suspected VAP and COVID-19, evaluating the diagnostic performance of the BioFire® FilmArray® Pneumonia Panel (FA-PP). Respiratory secretion samples underwent standard microbiological culture and FA-PP assays, and the results were compared.

**Results:**

We included 252 samples. The traditional culture method detected 141 microorganisms, and FA-PP detected 277, resulting in a sensitivity of 95% and specificity of 60%, with a positive predictive value of 68% and negative predictive value of 93%. In samples with high levels of genetic material (> 10^5 copies/mL), the panel had a sensitivity of 94% and specificity of 86%. In addition, 40% of the culture-negative samples had positive FA-PP® results, of which 35% had > 10^5 copies/mL of genetic material. The most prevalent bacteria were Gram-negative bacilli, followed by Gram-positive cocci. The panel identified 98 genes associated with antimicrobial resistance, predominantly extended-spectrum beta-lactamases (28%).

**Conclusion:**

The FA-PP is a sensitive assay for identifying bacteria causing VAP in patients with COVID-19, with a greater capacity to detect bacteria than the conventional method. The timely microbiological recognition offered by this panel could lead to optimized decision-making processes, earlier tailored treatment initiation, and improved antibiotic stewardship practices.

## Background

Ventilator-Associated pneumonia (VAP) is a burdensome issue for healthcare systems due to its high mortality, consequent demand for broad-spectrum antibiotics, and high costs [1]. A rapid microbiological and accurate diagnosis can lead to prompt selection of antibiotic treatment, improving patient outcomes and antimicrobial stewardship. Delays in the administration of effective therapy in patients with VAP are associated with increased mortality and hospital length of stay. Moreover, recognizing resistance patterns is essential to provide a better-targeted treatment [2].

Although empiric antibiotic treatment is essential to improve prognosis, excessive use of antibiotics threatens to reduce the effectiveness of these drugs to a great extent in the near future. Some studies show that pathogen rates vary in hospitalized patients with pneumonia, and etiological diagnosis is only achieved in 38% of cases [3, 4]. One meta-analysis evaluating 24 studies, including more than 4500 patients, detected bacterial pathogens in 73% of the subjects. However, other studies found detection rates lower than 10% [5, 6]. Approximately half of the antibiotic prescriptions are unnecessary or incorrect. This number dramatically increased during the pandemic as it became difficult to distinguish viral infection progression and true pneumonia. Furthermore, over-treating patients with broad-spectrum antibiotics can be harmful through gut flora damage and favoring drug resistance [1, 7].

Cultures are standard diagnostic methods for pathogen determination in VAP. However, it takes 48 to 72 h to get a result, delaying the opportunity to match the specific pathogen and its resistance pattern with empirical treatment. Therefore, the development and implementation of molecular diagnostic tests for pneumonia in the last decade represent an essential improvement in the microbiological diagnosis of pathological respiratory diseases [8].

The BioFire FilmArray® Pneumonia Panel (FA-PP) identifies multiple respiratory pathogens and resistance genes. It aims to detect 18 bacterial pathogens (eleven Gram-negative, four Gram-positive, and three atypical), nine viruses, and seven determinants of resistance (namely CTX-M, KPC, NDM, OXA 48-like, VIM, IMP, and MecA/C/MREJ). This novel and potentially useful feature consists of a nested multiplex PCR-based method that takes an average time of 60 min [6]. The panel has been proven to have a high diagnostic among different types of infection, including central line-associated bloodstream infections and hospital-acquired pneumonia [9–11]. However, the platform acquisition cost is a one-time cost of around $35,000 US dollars, and each sample processing costs $214 US dollars per patient [12].

Since the first cases of COVID-19, several authors have recognized the importance of bacterial and fungal superinfection in patients admitted to intensive-care units (ICUs) [13, 14]. Co-infections in COVID-19 have been described in previous studies, and the reported incidence varies greatly [15–17]. According to several reports, depending on the defined criteria, the heterogeneity of patients included, and the diagnostic methods, hospital-acquired infections are a late complication, occurring after a median of more than one week after hospitalization [15–17]. Bardi et al. found that the prevalence of lower respiratory tract infections in patients with COVID-19 was 33% [18]. In a more recent study, bacterial co-infection in critically ill COVID-19 patients occurred in 24.54% and 17.27% of patients evaluated using the multiplexed molecular assay and traditional cultures, respectively [19].

The Instituto Nacional de Enfermedades Respiratorias Ismael Cosio Villegas (INER) is a tertiary reference center for treating respiratory diseases in Mexico. As a strategy to handle the COVID-19 pandemic, since March 1, 2020, the Mexican Health Ministry has designated the transformation of some health institutions, including INER, as COVID-19 exclusive centers [20].

From February 2020 to March 31, 2021, 2,614 patients with severe and critical COVID-19 were treated at INER, all requiring mechanical ventilation. The reported rate of co-infections in 2020 was 59%, whereas, in 2021, this figure decreased to 34%, with 927 patients diagnosed with hospital-acquired pneumonia (HAP) [21]. This downward trend occurred probably due to the implementation of efficient infection control measures.

INER utilizes the FA-PP for microbiological diagnosis in patients with pneumonia. Although this panel could offer a rapid alternative to microbiological cultures for pathogen identification, data on its effectiveness on patients with VAP and COVID-19 is limited. Moreover, this topic has never been studied in the Mexican population.

The present study aimed to evaluate the diagnostic performance of the FA-PP compared to a traditional culture in detecting bacterial co-infections in COVID-19 patients with VAP attending at INER (Mexico City, Mexico).

## Methods

We conducted an observational, diagnostic performance cross-sectional study at a COVID-19 tertiary referral hospital in Mexico City, the National Institute of Respiratory Diseases (INER). We included all adult patients with an RT-PCR-confirmed SARS-CoV2 infection admitted between March 2020 to March 2021 who had a suspected bacterial VAP (defined as a Clinical Pulmonary Infection Score [CPIS] [22] > 6 points, > 48 h after intubation), and who had culture results and a requested pneumonia panel that was processed on the same day. Patients with only culture or panel results at the time of VAP suspicion and those with incomplete laboratory data were excluded.

Per the institution’s standard protocols, all patients with suspected VAP underwent microbiological sampling via bronchoalveolar lavage (BAL), bronchial aspirate (BA), tracheal aspirate (TA), or sputum culture. In addition, a separate sample was obtained and subjected to conventional microbial identification and FA-PP for each patient. During the COVID-19 pandemic, usual diagnostic practices such as BAL were limited due to their associated high risk of exposure for the operator. Therefore, INER utilized TA, BA, and sputum samples as valid alternative diagnostic methods.

The collection of TA, BA, and sputum was performed using a siliconized polyvinyl-chloride (PVC) probe with a closed endotracheal suction system assembled with a sterile polypropylene collector bottle following strictly aseptic principles. At INER, BAL is performed by a pulmonology specialist. Fiberoptic bronchoscopy is performed through a 50–60 cm-long flexible tube with about 5 mm in diameter. The distal end of the tube is inserted into a tracheostomy or an orotracheal intubation tube. To perform a BAL, it is necessary to pin the bronchial tip of the bronchoscope in the area to be analyzed and instill three aliquots of sterile saline (20–50 mL each, with a total of 100–150 mL) independently and aspirate the maximum possible amount of instilled liquid. All BALs were seeded without a microscopic evaluation. A sample was considered to contain a pathogenic microorganism when the development was equivalent to ≥ 10,000 CFU / ml.

The TA, BA, and sputum samples were examined under microscopy, and sample quality was determined according to the Murray-Washington criteria [23]. After Gram examination, the good-quality specimens, those with ≤ 10 squamous epithelium cells and > 25 leukocytes per low-power field, were grown in Blood, McConkey, and Chocolate agar. The grown specimens were incubated for 24 to 48 h at 37 °C. Culture media with no growth were deemed as negative or sterile, while those with growth of more than two types of colonies were classified as contaminated. Specimens that only grew microorganisms typically encountered in the upper respiratory tract were considered culture-negative. The culture was positive when culture density reached more than 10^5^ CFU/ml. Standard microbiological identification of positive cultures was performed using VITEK® 2 (bioMérieux, Marcy-l’Etoile, France) systems. This automated system identifies pathogens and tests susceptibility [24]. Concomitant pathogen identification with BioFire® FilmArray® Pneumonia Panel (bioMérieux, Marcy-l’Etoile, France) was performed. All steps followed the manufacturer’s instructions [25]. Culture and FA-PP samples were obtained simultaneously. The identified pathogens and antimicrobial resistance genes were reported.

Sociodemographic, clinical, and microbiological data were obtained from the institution’s electronic health record. These included temperature, oxygen saturation, and characteristics of secretions. In addition, radiological evaluation was performed using the MD Resolution system (INER image viewer). Patients with incomplete clinical data were excluded from the analysis.

Utilizing clinical and radiological information, a pulmonologist and a clinical infectious disease specialist classified the patients into two groups using CPIS: COVID-19 with suspected bacterial pneumonia and COVID-19 with no criteria for bacterial co-infection. CPIS [22] of > 6 points was defined as new progressive and persistent pulmonary infiltrates (> 24 h) by chest X-ray or chest CT scan and two or more of the following criteria: Fever or hypothermia, leukocytosis (12 × 10^9^ cells/L) or leukopenia (4 × 10^9^ cells/L), purulent lung secretions, reduction in PaO2/FiO2 > 15% (with no other apparent cause), a quantitative culture of a sample of pulmonary secretions, obtained by BA, TA, or sputum (> 10^5^ CFU/mL) or BAL (> 10^4^ CFU/mL), isolation of microorganisms from blood cultures, in the absence of another probable focus, in the 48 h before or after obtaining a respiratory sample (TA, BA, sputum, or BAL). If microorganisms were isolated from respiratory samples and blood cultures, to confirm that the identified bacteria were causative pathogens, they had to be microbiologically identical with the same antibiotic sensitivity pattern.

Statistical analysis was conducted using SPSS 24.0 (IBM, Armonk, New York, USA) and GraphPad Prism 9.5.0 (GraphPad Software, San Diego, USA). Medians with interquartile ranges and means with standard deviations were used for quantitative variables; frequencies and percentages were used for qualitative data. We assessed the diagnostic yield of the FA-PP compared to the diagnostic standard, microbiologic culture. Based on these, the following were calculated: sensitivity (true positive results divided by the sum of the true positive and false negative results), specificity (true negative results divided by the sum of true negative and false positive results), positive predictive value (PPV, true positives divided by the sum of the true positive and false positive results), negative predictive value (NPV, true negatives divided by the sum of the true negative and false negative results), positive likelihood ratio (LR+, sensitivity divided by 1-specificity), and negative likelihood ratio (LR-, 1-sensitivity divided by specificity). We calculated agreement between tests with Cohen’s kappa coefficient.

This study follows the Helsinki Declaration and has been approved by the Institutional Review Board of the National Institute of Respiratory Diseases (Comité de Ética en Investigación INER, study no. C47-21), informed consent was obtained from all participants prior to participation in this study.

## Results

**Fig. 1:**
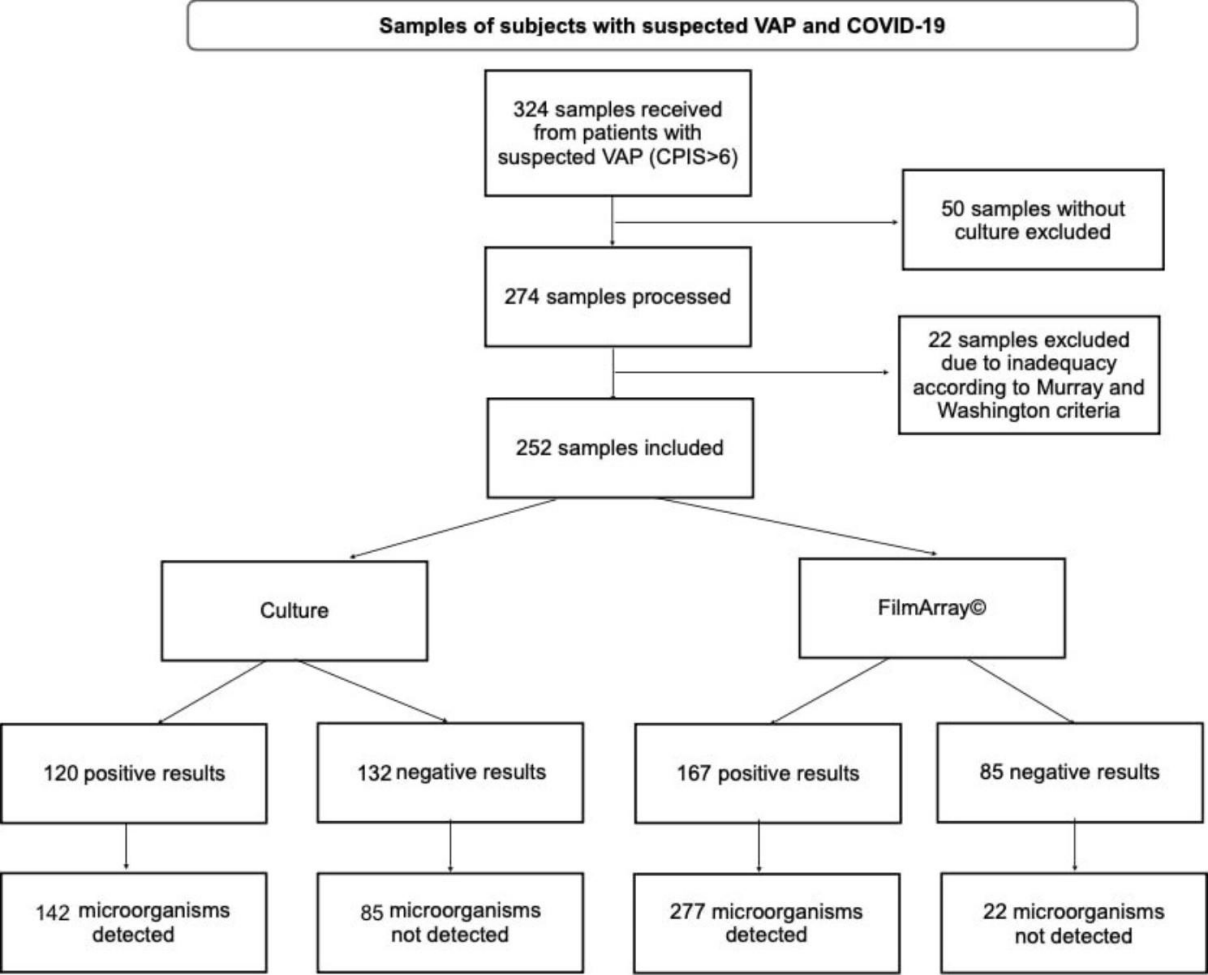
Samples of subjects with suspected Ventilator-Associated Pneumonia and COVID-19. The flow diaGram details the selection and assessment of sample eligibility for analysis. Abbreviations: VAP Ventilator-Associated Pneumonia, CPIS clinical pulmonary infection score

From March 15, 2020, to March 31, 2021, the clinical microbiology laboratory at INER performed 324 FA-PPs on subjects with confirmed COVID-19 infection and concomitant VAP. Of these samples, 50 were excluded from analysis due to lack of culture and 22 due to inadequacy based on the Murray Washington criteria (Fig. 1).

Most included subjects were men (n = 193, 77%), with a median age of 52 (IQR 34–65). In addition, comorbidities were frequent; the most prevalent was obesity (n = 98, 39%). All patients received invasive mechanical ventilation. Table 1 summarizes the baseline sociodemographic and clinical characteristics.

**Table 1:** Baseline patient characteristics

Sociodemographic data and comorbidities	
Characteristic	Total (n = 252)
Men, n (%)	193 (77%)
Age (years), median (IQR)	52 (34–65)
Obesity, n (%)	98 (39%)
Tobacco exposure, n (%)	79 (31%)
Hypertension, n (%)	74 (29%)
Diabetes mellitus, n (%)	91 (36%)
COPD, n (%)	6 (2%)
Asthma, n (%)	8 (3%)
HIV, n (%)	4 (2%)
**Clinical and laboratory data**	
Fever, n (%)	134 (53%)
Mucopurulent secretion, n (%)	78 (31%)
X-ray changes, n (%)	168 (67%)
Leukocyte count (10^3^/mm^3^), median (IQR)	12,75 (7,09–18,41)
Neutrophil count (10^3^/mm^3^), median (IQR)	10,9 (5,5–16,3)
Creatinine (mg/dL), median (IQR)	1,05 (0,79 − 2,49)
Procalcitonin, median (IQR)	1,76 (0,6–9,45)
PaO2/FiO2 ratio* <240 mmHg, n (%)	218 (87%)
PaO2/FiO2 ratio* (mmHg), median (IQR)	136,89 (85,33–190,94)
CPIS, median (IQR)	7 (6–9)
Days from mechanical ventilation to pneumonia onset, median (IQR)	17,68 (2–64,98)
Previous antimicrobial use, n (%)	202 (80%)

IQR interquartile range, COPD chronic obstructive pulmonary disease, CPIS Clinical Pulmonary Infection Score

*Relationship between the arterial oxygen partial pressure (PaO2) measured by arterial blood gas and the fraction of inspired oxygen (FiO2).

Most samples were (n = 243, 96%) obtained via bronchial aspirate. On macroscopic visualization, 78 (31%) had a mucopurulent appearance. Using culture and identification by the VITEK® 2 system -the reference method for bacterial detection-142 bacteria were identified from 120 (48%) positive samples. In contrast, the FA-PP identified 277 bacteria in 167 (66%) positive specimens. From the 252 samples, 114 (45.2%) were detected by both methods (with concordant bacterial identification), 53 (21%) were positive in FA-PP but negative in culture, 6 (2.4%) were detected by culture and were negative in FA-PP, and 79 (31.4%) were negative by both methods. As presented in Table 2, the FA-PP was 95% sensitive and 60% specific compared to culture, with a PPV of 68% and NPV of 93%. Cohen’s kappa coefficient for agreement was 0.53.

**Table 2:** Diagnostic performance of the BioFire® FilmArray® Pneumonia Panel compared to bacterial culture

BioFire® FilmArray® Pneumonia Panel*	Bacterial Culture (reference method)			Sensitivity (%)	Specificity (%)	PPV (%)	NPV (%)	LR (+)	LR (-)	κ
	Positive	Negative								
Positive	114	53		95	60	68	93	2.37	0.08	0.53
Negative	6	79								
Low and medium volumes of genetic material (less than 10^5^ copies/mL) *										
Positive	25		40	81	66	38	93	2.4	0.29	0.33
Negative	6		79							
High volumes of genetic material (more than 10^5^ copies/mL) *										
Positive	89		13	94	86	87	93	6.63	0.07	0.79
Negative	6		79							

PPV positive-predictive value, NPV negative-predictive value, LR likelihood ratio, κ Kappa for agreement

**Fig. 2:**
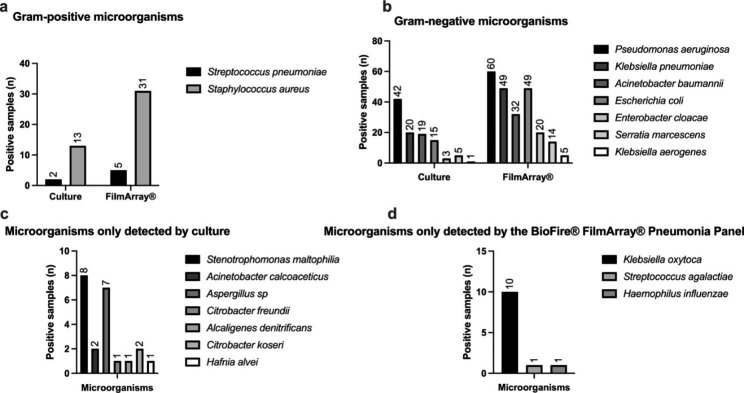
Comparison of microorganism detection between culture and the BioFire® FilmArray® Pneumonia Panel. **(a)** Gram-positive and **(b)** Gram-negative pathogens identified by both methods, **(c)** microorganisms only detected by culture, and **(d)** microorganisms only detected by the BioFire® FilmArray® Pneumonia Panel

The most frequently identified bacteria by both methods were *Pseudomonas aeruginosa, Klebsiella pneumoniae*, *Escherichia coli, Staphylococcus aureus*, and *Acinetobacter baumannii* (Table 3; Fig. 2). In the 53 cultures without pathogen detection in which FA-PP was positive, the system identified 85 bacteria; in 35% (n = 30) of these, the system reported high levels (> 10^5 copies/mL) of genetic material (Table 4). When analyzing diagnostic performance in samples with high levels of genetic material (> 10^5), sensitivity decreased to 94%, whereas specificity increased to 86%, with a kappa for agreement of 0.79 (Table 2).

**Table 3:** Microorganisms detected by culture and the BioFire® FilmArray® Pneumonia Panel

Microorganism	Culture n (%) n = 142(100)	BioFire® FilmArray® Pneumonia Panel n (%) n = 277 (100)
**Gram-negative**		
***Pseudomonas aeruginosa***	42 (30)	60 (22)
***Klebsiella pneumoniae***	20 (14)	49 (18)
***Acinetobacter baumannii***	19 (13)	32 (12)
***Escherichia coli***	15 (11)	49 (18)
***Enterobacter cloacae***	3 (2)	20 (7)
***Klebsiella aerogenes***	1 (1)	5 (2)
***Serratia marcescens***	5 (3)	14 (5)
***Klebsiella oxytoca***	0 (0)	10 (3)
***Haemophilus influenzae***	0 (0)	1(0)
**Gram-positive**		
***Streptococcus pneumoniae***	2 (1)	5 (2)
***Staphylococcus aureus***	13 (9)	31 (11)
***Streptococcus agalactiae***	0 (0)	1 (0)
**Outside panel spectrum**		
***Stenotrophomonas maltophilia***	8 (6)	Not detected
***Acinetobacter calcoaceticus***	2(1)	Not detected
***Aspergillus sp***	7 (5)	Not detected
***Citrobacter freundii***	1(1)	Not detected
***Alcaligenes denitrificans***	1(1)	Not detected
***Citrobacter koseri***	2(1)	Not detected
***Hafnia alvei***	1(1)	Not detected

**Table 4:** Microorganisms identified by the BioFire® FilmArray® Pneumonia Panel in the 53 culture-negative samples

Microorganism	Total (n = 85)	Less than 10^5 copies/mL (n = 55)	More than 10^5 copies/mL (n = 30)
*Klebsiella pneumoniae*	21	13	8
*Pseudomonas aeruginosa*	14	7	7
*Escherichia coli*	13	8	5
*Acinetobacter baumannii*	13	9	4
*Staphylococcus aureus*	6	3	3
*Enterobacter cloacae*	5	4	1
*Klebsiella oxytoca*	10	8	2
*Serratia marcescens*	2	2	0
*Streptococcus pneumoniae*	1	1	0

**Fig. 3:**
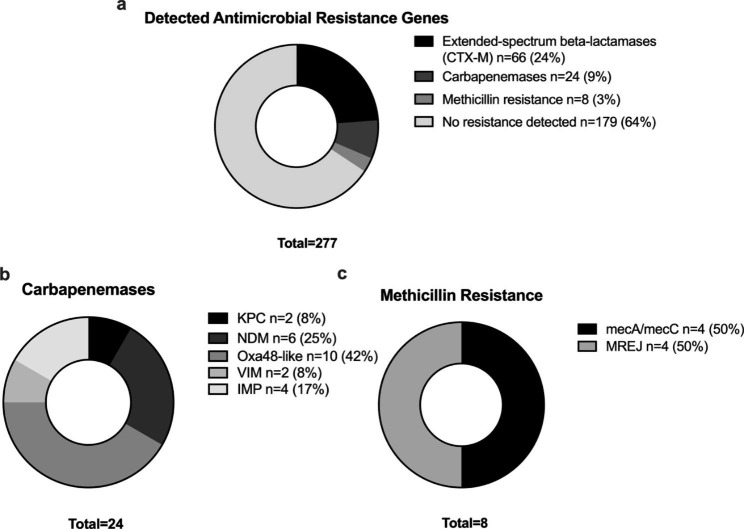
Resistance genes identified by the BioFire® FilmArray® Pneumonia Panel. The figure displays **(a)** the overall prevalence of resistance genes among the identified microorganisms (n = 277), **(b)** carbapenemases detected in Gram-negative bacteria (n = 240), and **(c)** methicillin resistance genes in samples with *S. aureus* identification (n = 31)

As a secondary analysis, we describe the detected antimicrobial resistance (AMR) genes by the FA-PP. The panel identified 98 genes associated with AMR, of which extended-spectrum beta-lactamases CTX-M were the most prevalent (n = 66/240, 28%) for the Gram-Negative microorganisms. In addition, the FA-PP panel detected carbapenemases in 10% (n = 24/240) of Gram-Negative bacteria, mostly class D (Oxa48-like [n = 10/24, 42%], followed by class B (NDM [n = 6/24, 25%], IMP (n = 4/24, 17%], and VIM [n = 2/24, 8%]), and class A (KPC [n = 2/24, 8%]). The methicillin-resistant Staphylococcus aureus rate was 25% (n = 8/31) (Fig. 3).

## Discussion

In this study, the FA-PP effectively identified a pathogen in more cases than traditional culture methods in the same BAL sample, detecting positive results in 66% and 48% of cases, respectively. Moreover, the FA-PP exhibited a broader detection of pathogens, finding 277 microorganisms versus 141 by traditional cultures. Nevertheless, the FA-PP was less specific than the culture, requiring interpretation based on careful clinical judgment and correlation with patients’ clinical picture. FA-PP was 95% sensitive and 60% specific compared to culture, with PPV of 68% and NPV of 93%. These parameters slightly differ from the Young Yoo study, which demonstrated an overall agreement on the results of FA-PP with the routine culture of 79.0%, with sensitivity and specificity of 98.5% and 76.5% for the FA-PP test, respectively [26]. Similarly, a single-center laboratory diagnostic study comparing culture results versus FA-PP showed that the latter detected substantially more pathogens [27]. A multicentre randomized trial conducted in the UK, where adults and children with suspected VAP or HAP were randomized to standard care culture or FA-PP, revealed in initial results that 76.5% of participants undergoing FA-PP were considered to receive adequate and proportionate antibiotics at 24 h, versus 55.9% in culture group. Moreover, at 72 h, this rate was 73.4% and 58.8%, respectively. Nonetheless, the clinical cure rate of pneumonia 14 days after diagnosis was 56.7% in the FA-PP and 64.7% in the culture group. ICU length of stay did not differ between groups [28]. A recent prospective cohort study performed in Egypt showed an overall sensitivity and specificity of FA-PP as high as 100% and 90%, respectively, in patients with HAP [10]. The evidence presented in these studies aligns with our findings, providing further support for the remarkable diagnostic accuracy of the panel.

The results of our study revealed that 21% of the samples that were negative by culture were positive when processed by FA-PP, posing the question of whether they were false positives or whether the panel’s performance was more accurate than culture. Two main factors could account for this discrepancy: First, the semiquantitative positivity threshold set by the manufacturer is > 10^3.5 copies/mL, which could decrease the specificity and PPV of the test, as evidenced in our stratified analysis. However, when only considering samples with > 10^5 copies/mL as positive, the specificity increased from 60 to 86% without altering sensitivity. This result is comparable to that conducted by Ferrer et al., which described a median bacterial load of > 10^7 copies/mL in positive FA-PP samples in a similar population. This change could be plausible and increase confidence in clinical decision-making by reducing false-positive rates. Second, many patients in our study received antibiotic therapy before sampling, which could deliver inconsistent results [29]. The results of the panel should be carefully interpreted and correlated clinically because false-positive samples could account for the unwarranted treatment of bacterial colonization rather than VAP.

The FA-PP categorizes samples based on the detection of genetic material, residual nucleic acids -without active bacterial replication because of antimicrobial effects- can be detected as positive by syndromic panels, as described in previous studies [26, 30]. This feature of molecular assays could be helpful by allowing a prompter treatment initiation without altering test results. To clarify this discrepancy, further microbiologic diagnostic techniques such as sample sequencing could be performed.

The results of this study are comparable to a prospective cohort study conducted in a tertiary care center in Mexico City, which was converted into a COVID-19 hospital. Among VAP cases in patients with COVID-19, enterobacteria were the most found microbial isolate, followed by non-fermenting Gram-negative bacilli and polymicrobial infections [31]. Furthermore, in this study, antimicrobial resistance was observed in around 50% of VAP episodes, predominantly caused by beta-lactamases, particularly AmpC producers [31], similar to our research, in which the most prevalent resistance gene was for the CTX-M beta-lactamase.

Although other studies have been carried out in lower and middle-income settings [9, 32], to the best of our knowledge, this was the first study conducted in the Mexican population to compare the diagnostic performance between FA-PP versus standard culture.

The FA-PP could impact the prescription of antibiotics in 70.7% of the patients, with interruption or reduction in 48.2% of the patients, thus contributing to an average saving of 6.2 antibiotic days/patient [33]. Therefore, the objective of using this diagnostic test is timely detection and rational use of antibiotics with all the impact on mortality and costs that it entails [8, 34]. Ferrer et al. reported a cost-benefit analysis of implementing FA-PP as an early diagnostic method and considering the economic burden imposed by antimicrobial therapy changes. Among 99 patients who were started with empirical antimicrobial therapy before FA-PP results, having selected appropriate empirical antimicrobial therapy since the beginning of treatment would have saved 6675€ [29].

Limitations of this study include the small sample size and the cross-sectional nature of its design. Further research should address the correlation between quantitative culture results and the number of copies of genetic material found in the FA-PP. Moreover, examining clinical outcomes, length of stay, and costs in VAP patients treated with a pathogen and resistance-matched antibiotic after FA-PP could be contrasted with VAP patients receiving empiric antibiotic treatment, considering a larger sample of patients.

## Conclusions

Molecular methods can detect microorganisms resulting in a high sensitivity compared to conventional methods. Our study found that the FA-PP system is a fast, sensitive method with a greater capacity to detect bacteria that cause lower respiratory tract infections compared to the standard method of detection. Although not yet able to replace conventional culture, these panels could be helpful in the hospital setting, allowing for faster microorganism and antimicrobial resistance detection and leading to improved antibiotic stewardship and decision-making processes.

## Data Availability

All data generated or analyzed during this study are included in this article. Further inquiries can be directed to the corresponding author.

## List of Abbreviations

VAP: Ventilator-Associated pneumonia
FA-PP: BioFire® FilmArray® Pneumonia Panel
IQR: Interquartile range
COPD: Chronic obstructive pulmonary disease
CPIS: Clinical Pulmonary Infection Score
PPV: Positive predictive value
NPV: Negative predictive value
LR: Likelihood ratio
ICU: Intensive-care unit
RT-PCR: Reverse transcriptase polymerase chain reaction
INER: Instituto Nacional de Enfermedades Respiratorias
BAL: Bronchoalveolar lavage
TA: Tracheal aspirate
BA: Bronchial aspirate
HAP: Hospital-acquired pneumonia
